# Circulating MicroRNAs and Long Non-coding RNAs as Potential Diagnostic Biomarkers for Parkinson’s Disease

**DOI:** 10.3389/fnmol.2021.631553

**Published:** 2021-03-04

**Authors:** Yimin Yang, Yanhua Li, Hongmei Yang, Jianxing Guo, Nan Li

**Affiliations:** Department of Intensive Care Unit, The First Hospital of Jilin University, Changchun, China

**Keywords:** Parkinson’s disease, circulating, biomarkers, miRNA, lncRNA

## Abstract

Parkinson’s disease (PD) is the world’s second most common neurodegenerative disease that is associated with age. With the aging of the population, patients with PD are increasing in number year by year. Most such patients lose their ability to self-care with disease progression, which brings an incalculable burden to individual families and society. The pathogenesis of PD is complex, and its clinical manifestations are diverse. Therefore, it is of great significance to screen for circulating biomarkers associated with PD to reveal its pathogenesis and develop objective diagnostic methods so as to prevent, control, and treat the disease. In recent years, microRNAs (miRNAs) and long non-coding RNAs (lncRNAs) are considered to be effective biomarkers for various diseases due to their stability, and resistance to RNAase digestion and extreme conditions in circulating fluids. Here, we review recent advances in the detection of abnormally expressed miRNAs and lncRNAs in PD circulating fluids, and discuss the function and molecular mechanisms of plasma or serum miR-124, miR-132, miR-29, miR-221, miR-7, miR-433, and miR-153 in the regulation and progression of PD. Additionally, application of the differential expression of lncRNAs in circulating fluid in the pathological progression and diagnosis of PD is also reviewed. In short, the determination of abnormally expressed circulating miRNAs and lncRNAs will be valuable for the future diagnosis and treatment of PD.

## Introduction

Parkinson’s disease (PD) is the world’s second most common neurodegenerative disease associated with aging, with an incidence close to 1% in people over 60 years of age (Elbaz et al., 2016). Its onset involves the interaction of genetic and high-risk factors acquired from environment processes (Kalia and Lang, 2015). At present, it is believed that motor dysfunction, such as rigidity, postural instability, tremor and bradykinesia, is related to a decrease in dopaminergic neurons in the striatum (Tysnes and Storstein, 2017). This is also one of the most prominent pathological features of PD. The abnormal deposition of alpha synuclein in Lewy bodies leads to the degeneration of dopamine cells (Surmeier et al., 2017). With the aging of the population, the number of people with PD is increasing on a yearly basis. Most patients, however, lose their ability to self-care with progression of disease, which brings an incalculable burden on families and society (Ascherio and Schwarzschild, 2016).

Although PD has long been the focus of research in the field of neurodegenerative diseases, making a diagnosis of PD still faces many difficulties (Cacabelos, 2017) due to: (1) An unclear pathogenesis: PD is affected by a variety of factors. Degeneration of dopaminergic neurons in patients with PD may be related to a series of mechanisms such as oxidative stress (Puspita et al., 2017), neurotrophic factor deficiency (Ledeen and Wu, 2018), excitotoxicity (Chen et al., 2019; Sekar and Taghibiglou, 2020), mitochondrial dysfunction (Kumari et al., 2020), abnormal immune regulation, and apoptosis (Wang L.Y. et al., 2019). In addition, at least six hereditary single gene mutations have been shown to be associated with the onset of PD (Gan-Or et al., 2015). (2) A lack of objective laboratory diagnostic methods: PD is highly heterogeneous. Currently, in addition to imaging methods, unified Parkinson’s and Hoehn–Yahr scales are common methods for the clinical diagnosis and evaluation of PD (Guan et al., 2019). However, due to the complicated pathogenesis of this disease, clinical symptoms and signs between different patients may be distinct, with impaired motor functions usually occurring a few years after the onset of disease. Moreover, despite considerable efforts, clinical biomarkers have not been identified as yet (Kalia and Lang, 2015). Therefore, screening for PD-related biomarkers is of great significance in revealing disease pathogenesis and developing objective diagnostic methods to prevent and treat disease. This review analyzes the relationship between ectopically expressed micro(mi)RNAs and long non-coding (lnc)RNAs in circulating fluids and patients with PD, and provides strong evidence for finding new PD biomarkers and therapeutic targets.

## Circulating miRNAs and PD

### Circulating miRNAs

MicroRNAs are endogenous single-stranded short sequence non-protein coding RNAs, 19–23 nucleotides in length. They are widely present in eukaryotes, are important regulatory molecules that regulate the expression of other functional genes, and are involved in regulating many cell physiological processes (Tafrihi and Hasheminasab, 2019). In mature and immature brains, miRNAs are expressed at very high levels in different brain regions and cell types, with certain miRNAs appearing in synapses, dendrites, and axons. MiRNAs have also been shown to be tissue-enriched, including different regions of the brain. Cogswell et al. (2008) investigated the direction and concentration of miRNA expression in the hippocampus, medial frontal gyrus and cerebellum in patients with Alzheimer’s disease (AD). They revealed the dysregulation of region-specific and Braak stage-specific miRNAs (Cogswell et al., 2008). Correspondingly, Bekris et al. (2013) studied the relationship between brain miRNAs, plasma and CSF in patients with AD at different Braak stages. Their study showed that miRNAs were specific to brain regions. These results suggested that the changes in miRNA levels in circulating biological fluids may be related to the changes in miRNA levels in the brain. Therefore, the characteristics of circulating miRNA can reflect the physiological and pathological conditions of different CNS diseases (Bekris et al., 2013). The secretion of miRNAs is selective and can change due to different pathological processes. Several researchers have found that miRNAs exist in human serum, plasma, urine, tears, saliva, amniotic fluid, and semen, and show different expression profiles. The expression of miRNAs in body fluids is not affected by changes in endogenous substances, and exogenous physical and chemical conditions (Glinge et al., 2017; Sanz-Rubio et al., 2018). In addition, circulating miRNAs can be quantitatively determined by different technologies, such as next-generation sequencing (NGS), quantitative real-time polymerase chain reaction (qRT–PCR), and microarray technology. Their ease of detection suggests broad prospects to diagnostic biomarkers for disease. The use of cerebrospinal fluid (CSF) is an advantage because it interacts closely with the brain. Therefore, it is expected that circulating miRNAs in CSF are similar to brain miRNAs. However, the risk of bleeding and infection in patients subjected to lumbar puncture for CSF sampling means this is procedure is not readily accepted by patients (Starhof et al., 2019). The study of miRNAs in blood is a good example of a minimally invasive method for the early diagnostic and prognostic evaluation of patients with PD. Van Den Berg et al. (2020) suggested circulating miRNAs have the potential to become highly valuable biomarkers for central nervous system (CNS) disorders, including PD. However, their study included only a small number of miRNAs associated with PD. Schulz et al. (2019) performed a comprehensive meta-analysis of dysregulated miRNAs in patients with PD; although their study was carried out in accordance with strict standards as a meta-analysis, several limitations still existed. For example, the included study lacked accurate *P*-values; the potential presence of publication bias and/or selective reporting bias was also inevitable (Schulz et al., 2019). Below, we introduce blood and CSF miRNAs as potential biomarkers for a PD diagnosis.

### Blood miRNAs and PD

**FIGURE 1:**
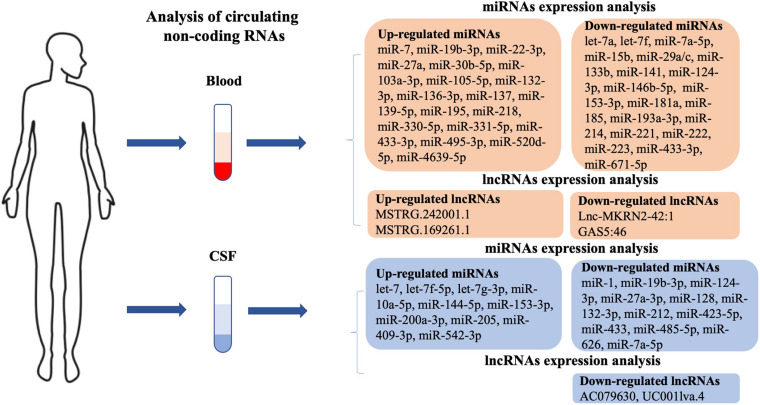
Potential circulating biomarkers in PD patients. Blood and cerebrospinal fluid (CSF) biomarkers include dysregulated micro (mi)RNAs and long non-coding (lnc)RNAs. PD, Parkinson’s disease.

Using circulating miRNAs in blood as a large-scale peripheral biomarker of PD in patients is a simple, economical, minimally invasive, and time-saving method of detection. Emerging studies have shown the differential expression of miRNAs in blood between patients with PD and healthy controls. The expression levels of miRNAs, including *Homo sapiens* (hsa)-miR-7-5p, hsa-miR-22-3p, hsa-miR-136-3p, hsa-miR-139-5p, hsa-miR-330-5p, hsa-miR-433-3p, hsa-miR-495-3p (Ravanidis et al., 2019), hsa-miR-27a (Chen et al., 2018), hsa-miR-137 (Li et al., 2017), hsa-miR-331-5p (Cardo et al., 2013), hsa-miR-19b-3p (Uwatoko et al., 2019), hsa-miR-520d-5p (Jin et al., 2018), hsa-miR-195 (Ding et al., 2016), hsa-miR-29a-3p, hsa-miR-30b-5p, hsa-miR-103a-3p (Serafin et al., 2015), and hsa-miR-105-5p (Yang et al., 2019a), were up-regulated, while hsa-let-7a, hsa-let-7f, hsa-miR-142-3p, hsa-miR-222 (Chen et al., 2018), hsa-miR-433, hsa-miR-133b (Zhang et al., 2017), hsa-miR-671-5p (Uwatoko et al., 2019), hsa-miR-141, hsa-miR-214, hsa-miR-146b-5p, hsa-miR-193a-3p (Dong H. et al., 2016), hsa-miR-29 (Bai et al., 2017), hsa-miR-15b, hsa-miR-181a, hsa-miR-185, hsa-miR-221 (Ding et al., 2016), hsa-miR-153, hsa-miR-223 (Cressatti et al., 2019), and hsa-miR-133b (Zhao et al., 2014) were found to be down-regulated in the blood of patients with PD compared with that of healthy controls (Figure 1). Hsa-miR-7 is down-regulated in the peripheral blood and regulates the expression of brain-derived neurotrophic factor (BDNF) through an auto-regulatory mechanism. Li et al. (2019) suggested that hsa-miR-7 regulates the BDNF axis in the early stages of PD and could serve as a biomarker for PD treatment (Li et al., 2019). Hsa-miR-218 is significantly up-regulated and regulates SLC6A3, TH, and EBF3, which may be involved in the pathogenesis of PD and therefore may be therapeutic strategies for PD (Li et al., 2018a). Hsa-miR-4639-5p is abnormally up-regulated and down-regulates *PARK7*, a well-known PD-related gene, further leading to severe oxidative stress and neuronal death (Chen et al., 2017). The review of prior studies suggests that several circulating miRNAs play a central role in pathways that lead to PD. Next, we detail such miRNAs that have been studied more intensively in the progression of PD.

### miR-124-3p and PD

The expression of mmu-miR-124-3p is abundant in dopaminergic neurons, with the expression level decreasing in a 1−methyl−4−pheny−1,2,3,6−tetrahydropyridine (MPTP) model of PD (Wang et al., 2016). Up-regulated mmu-miR-124-3p significantly reduced dopaminergic neuron loss by regulating apoptosis and attenuating an impaired autophagic process in MPTP-treated mice. Mmu-miR-124-3p has potential neuroprotective properties. Its protective mechanism may regulate the expression of calpain/cdk5 pathway protein in dopaminergic neurons by inhibiting Bim, and reduces the damage of apoptosis and autophagy in dopaminergic neurons by reducing the transfer of Bax to mitochondria and lysosomes (Kanagaraj et al., 2014). Another possible neuroprotective role of mmu-miR-124-3p is to enhance the viability of 6-hydroxydopamine (6-OHDA)-treated PC12 or SH-SY5Y cells by targeting annexin A5, which is associated with stimulation of the ERK pathway (Dong et al., 2018). Mmu-miR-124-3p inhibits neuroinflammation in PD by targeting p62, p38, and autophagy, suggesting that mmu-miR-124 might be a potential therapeutic target for modulating the PD inflammatory response (Yao et al., 2019).

The plasma hsa-miR-124 concentration has been shown to be a promising biomarker for cerebral infarction, although whether the authors detected miR-124-3p or miR-124-5p was not specified in this study (Weng et al., 2011). In view of its important role in the pathogenesis of PD, it is reasonable to suggest that it can be used as a potential biomarker for PD diagnosis and progression. A recent study that enrolled 60 PD patients and 60 healthy controls showed that plasma hsa-miR-124-3p levels were significantly lower in patients with PD (an area under the receiver operating characteristic curve of 0.709) than that of healthy controls. However, as assessed by the Unified Parkinson’s Disease Rating Scale (UPDRS), Hoehn and Yahr scales, a statistically significant association was not found between hsa-miR-124-3p levels and the severity of motor symptoms, possibly due to the small sample size (Li et al., 2017). Therefore, hsa-miR-124-3p may serve as a diagnostic biomarker for PD, although further studies require a larger sample size.

### miR-132-3p and PD

MiR-132-3p targets DNA methylation, long-term potentiation, neuronal cAMP response element binding protein, BDNF signal transduction, and the N-methyl-D-aspartate receptor, and participates in important approaches to neural metaplasticity (Qian et al., 2017). Previous studies have reported that *Rattus norvegicus* (rno)-miR-132-3p was up-regulated in the striatum of the parkinsonian rat (Horst et al., 2018) as well as in a transgenic model of PD (Lungu et al., 2013). Nurr1, as a transcription factor, plays a key role in the differentiation and functional maintenance of mesencephalic dopaminergic neurons (Saijo et al., 2009; Dong J. et al., 2016). Yang et al. demonstrated that over-expression of hsa-miR-132-3p repressed the differentiation of embryonic stem cells into dopaminergic neurons by directly targeting the *Nurr1* gene (Yang et al., 2012).

Yang et al. (2012) demonstrated that the plasma hsa-miR-132-3p level in PD was significantly higher than in healthy and neurological disease controls. Moreover, a higher level of hsa-miR-132-3p expression was closely associated with a risk for PD in males, and was related to disease severity and stage (Yang et al., 2019b). Van Den Berg et al. (2019) concluded that expression levels of hsa-miR-132-3p were three times higher in the peripheral blood lymphocytes of treated patients with PD. Thus, it was suggested that hsa-miR-132-3p might be a potential biomarker for the diagnosis of patients with PD and their response to treatment (Van Den Berg et al., 2019).

### miR-29 and PD

The miR-29 family, including four mature miR sequences (miR-29a, miR-29b-1, miR-29b-2, and miR-29c) (Goh et al., 2019), has been reported to play critical roles in neurodegenerative diseases, including PD (Wang et al., 2014). Previous studies have shown that miR-29a/c has important roles in PD development, including immune regulation, fine-tuning motor functions (Papadopoulou et al., 2015; Bai et al., 2017), epigenetic modulation (Lyu et al., 2018), neuronal survival (Roshan et al., 2014), and apoptosis (Kole et al., 2011). MiR-29a regulated the immune response by targeting T-helper 1 differentiation and regulating transcription factor T-bet expression, thereby inhibiting IFN-γ production (Steiner et al., 2011). MiR-29b was activated during neuronal maturation and restricted apoptosis by targeting *BH3* gene expression (Kole et al., 2011).

Expression levels of the miR-29 family were significantly down-regulated in the serum of patients with PD. Bai et al. (2017) reported that the expression levels of hsa-miR-29a and hsa-miR-29c were down-regulated more significantly than hsa-miR-29b. In addition, hsa-miR-29a and hsa-miR-29c expression levels were negatively associated with PD severity. Moreover, these two miRs in serum were higher in female patients with PD, suggesting that the miR-29 family expression level might be gender-specific (Bai et al., 2017). The hsa-miR-29a level was found to be significantly decreased in the peripheral blood of patients with PD, but was unrelated to L-dopa treatment (Margis et al., 2011). Botta-Orfila et al. (2014) concluded that hsa-miR-29a/c was down-regulated and that this might be related to gender. However, age was not associated with the level of hsa-miR-29a/c in patients with PD when compared with controls (Botta-Orfila et al., 2014). Bai et al. (2017) also suggested that decreased serum levels of hsa-miR-29a and hsa-miR-29c were related to PD severity. Gui et al. (2015) showed that the hsa-miR-29c expression level was decreased in exosomes isolated from the CSF of patients with PD. These studies suggest that the dysregulation of hsa-miR-29a/c might mediate PD progression and be a promising biomarker for the diagnosis of PD.

### miR-221-3p and PD

The expression of mmu-miR-221-3p was reported to be down-regulated in a PC12 cell model of PD. Li et al. (2018b) determined that a mmu-miR-221-3p mimic could significantly inhibit apoptosis and promote the viability and proliferation of 6-OHDA–treated PC12 cells (Li et al., 2018b). Hsa-miR-221-3p also showed decreased expression in MPP^+^-treated SH-SY5Y cells (Asci et al., 2013). Previous studies have demonstrated that iron accumulation has a potential role in PD pathogenesis, and that mutations in genes involved in iron homeostasis are related to a higher risk of developing PD (Borie et al., 2002; Guerreiro et al., 2006). The TFR2a receptor expressed on the cell membrane is involved in iron uptake, while PD is characterized by iron overload (Andolfo et al., 2010). Asci et al. (2013) showed that TFR2 expression was regulated by hsa-miR-221-3p in a cellular model of PD. An inverse correlation existed between TFR2 and hsa-miR-221-3p in MPP^+^-treated SHSY5Y cells (Asci et al., 2013). Salama et al. (2020) concluded that the enhanced expression rno-miR-221-3p contributed to Akt/mammalian target of rapamycin (mTOR) activation, which suggested that rno-miR-221-3p played a protective role in PD and may serve as a potential therapeutic target for PD treatment.

Ma et al. (2016) measured the expression of 16 miRNAs in the sera of 138 PD patients and 112 healthy controls by qRT–PCR. They found hsa-miR-221 was significantly decreased in patients with PD; the receiver operating characteristic result of serum hsa-miR-221 for a prediction of PD was 0.787. In addition, serum hsa-miR-221 was positively correlated with UPDRS-III and -V scores in PD patients, indicating that down-regulated serum hsa-miR-221 might be a potential biomarker for the evaluation of PD, although whether the authors detected hsa-miR-221-3p or hsa-miR-221-5p was not specified in this study (Ma et al., 2016).

### miR-7a-5p and PD

MiR-7a-5p is a conserved gene in the CNS that is known to regulate synaptic plasticity and neuronal differential (Doxakis, 2010). The decreased expression of mmu-miR-7a-5p in MPTP-treated mice leads to the elevated expression of *SNCA* and degeneration in the nigrostriatal system (McMillan et al., 2017). The expression level of rno-miR-7a-5p in blood and brain tissue were found to be in opposing directions. Li et al. (2019) suggested that the rno-miR-7a-5p level was up-regulated in the SNpc but down-regulated in blood. Besides post-transcriptionally regulating α-synuclein (α-syn) expression in dopaminergic neurons, which is involved in the pathophysiology of PD, it was found that rno-miR-7a-5p also bound to the 3′ untranslated region (UTR) of the BDNF transcript, a critical factor involved in signaling and synaptic plasticity in the CNS. Down-regulated rno-miR-7a-5p stimulated the expression of BDNF, which activates protective mechanisms in dopaminergic neurons. Fragkouli and Doxakis (2014) found that mmu-miR-7a-5p repressed α-syn formation, and indirectly protected dopaminergic neurons by up-regulating the mTOR pathway (Fragkouli and Doxakis, 2014). Zhou et al. (2016) concluded that mmu-miR-7a-5p also targeted *Nlrp3* expression and inhibited activation of the NOD-like receptor pyrin domain-containing protein 3 inflammasome in PD mice. Decreased serum levels of hsa-miR-7a-5p were also observed in 12 patients with PD compared to healthy controls (Zhou et al., 2016). Collectively, these results indicate the possible contribution of hsa-miR-7a-5p and its target genes to the development of the complex pathophysiology of PD, and also open up a promising therapeutic avenue for PD.

### miR-433 and PD

MiR-433 is highly expressed in the CNS (Davis et al., 2005). Several previous studies have identified fibroblast growth factor 20 (FGF20) as a risk factor. *FGF20* has been suggested as one of the candidate genes responsible for PD in Chinese and Japanese populations (Pan et al., 2012). The increased translation of *FGF20* was related to increased α-syn expression, which was shown to lead to the development of PD (Ma et al., 2015). Tarale et al. (2018) proposed hsa-miR-433-3p that bound to FGF20 mRNA transcripts negatively regulated FGF20 protein translation. Their study explored the regulatory relationship between hsa-miR-433-3p and *FGF20*, which might be potentially useful for PD diagnosis and treatment. The expression of hsa-miR-433-3p was also significantly decreased in SH-SY5Y cells. Tarale et al. demonstrated that *FGF20* was a target of hsa-miR-433-3p with an inverse correlation in expression levels (Tarale et al., 2018).

Zhang et al. (2017) compared 46 sporadic PD patients and 49 healthy controls by collecting miRNA profiles of plasma samples. The study observed that circulating hsa-miR-433-3p was significantly reduced in PD. These results suggested that aberrant expression of hsa-miR-433-3p might be involved in the pathophysiology of PD by regulating α-syn expression-dependent processes in the brain. Consequently, the plasma hsa-miR-433-3p level may serve as a diagnostic biomarker for PD.

### miR-153-3p and PD

Both hsa-miR-153-3p, as well as α-syn mRNA and protein, are present in high levels in brain tissues, such as the midbrain, cortex, and hippocampus, and their expression is restricted to neurons (Mouradian, 2012; Cressatti et al., 2019). Hsa-miR-153-3p is a miRNA found in the brain that has been shown to post-transcriptionally regulate α-syn expression levels (Chu and Kordower, 2007). Doxakis (2010) showed that α-syn was directly targeted by mmu-miR-153-3p by binding to the 3′-UTR of α-syn mRNA with an inverse correlation. They further speculated that overexpression of mmu-miR-153-3p significantly suppressed α-syn expression, promoted neurite outgrowth, and was neuroprotective against classical PD insults in tissue culture models (Doxakis, 2010; Kim et al., 2013). Except for modulating α-syn expression, mmu-miR-153-3p protects neurons exposed to PD insults by altering intracellular signaling. Fragkouli and Doxakis (2014) demonstrated how mmu-miR-153-3p protected cortical neurons against MPP^+^-induced toxicity by preserving the activation of the downstream master integrating signaling pathway of mTOR. It was concluded that overexpression of mmu-miR-153-3p activated the mTOR pathway to significantly increase its downstream effectors, which suggested miR-153-3p might act as an “activator” of this pathway. Additionally, signaling pathways of SAPK/JNK and p38 in MPP^+^-treated cells might also be modulated by mmu-miR-153-3p (Fragkouli and Doxakis, 2014).

Zhang et al. (2017) indicated that the plasma hsa-miR-153-3p level was significantly reduced in a PD patient. Cressatti et al. (2019) explored salivary hsa-miR-153-3p levels of patients with PD compared with non-neurological controls. The study revealed that hsa-miR-153-3p salivary levels decreased, although miRNA expression levels did not change with disease progression (Hoehn and Yahr stages) (Cressatti et al., 2020). Collectively, existing results suggested the possible contribution of hsa-miR-153-3p in the development of PD. Thus, the hsa-miR-153-3p level may serve as a useful, non-invasive diagnostic biomarker of idiopathic PD.

### CSF miRNAs and PD

Because of its direct contact with the CNS, an analysis of CSF can accurately reflect CNS biochemical changes; it is also a readily accessible body fluid specimen. Dysregulated miRNAs in CSF may be the reflection of miRNA expression in the brain and provide an experimental basis for such miRNAs to be used to assist in the laboratory diagnosis and efficacy observation index of PD, as well as therapeutic targets for PD treatment. In practice, CSF is rarely collected because it is more invasive than blood sampling (Van Den Berg et al., 2020). Nevertheless, in recent years, increasing numbers of studies have shown that CSF contains differentially expressed miRNAs, which are important biomarkers with high prediction accuracy during the onset of PD (Figure 1). Expression levels of hsa-let-7 (Zhao et al., 2019), hsa-miR-10a-5p, hsa-miR-409-3p, hsa-let-7g-3p (Gui et al., 2015), and hsa-let-7f-5p (Dos Santos et al., 2018) were elevated in the CSF of patients with PD when compared with healthy controls. Cho et al. (2013) observed a lower expression of hsa-miR-205 in the brain regions of 15 patients diagnosed with PD (Cho et al., 2013). In contrast, Gui et al. (2015) found that the expression level of hsa-miR-205 was significantly high in the CSF of patients with PD. The expression levels of hsa-miR-144-5p, hsa-miR-200a-3p, and hsa-miR-542-3p were significantly elevated in both A53T-transgenic mice and patients with PD, being accurate for the prediction of PD. In addition, logistic regression analysis revealed that the severity of PD showed a positive correlation with up-regulated hsa-miR-144-5p, hsa-miR-200a-3p, and hsa-miR-542-3p levels in CSF (Mo et al., 2017). Expression levels of hsa-miR-128, hsa-miR-132, hsa-miR-212, hsa-miR-433, hsa-miR-485-5p (Burgos et al., 2014), hsa-miR-1, hsa-miR-19b-3p (Gui et al., 2015), hsa-miR-27a-3p, and hsa-miR-423-5p (Dos Santos et al., 2018) were decreased in the CSF of patients with PD. Marques et al. (2017) found that the level of hsa-miR-24 was decreased in the CSF of patients with PD when compared to healthy controls. However, the result was in contrast to that previously obtained by Vallelunga et al. (2014) who suggested hsa-miR-24 occurred at higher levels in PD serum (Vallelunga et al., 2014). Few exploratory studies have compared plasma/serum and CSF circulating miRNA profiles directly in the same cohort. This discrepancy may be due to differences in the body fluids used (serum vs. CSF), where CSF is more closely related to neurodegeneration, while serum levels may represent systemic changes. In addition, the difference in the direction of changes in circulating miRNA concentrations may have a physiological reason or be due to pathological changes. Moreover, blood volume is greater than CSF volume; therefore, the concentration of circulating miRNAs in blood may be diluted compared to CSF. MiR-626 is highly expressed in SNpc (Tsitsiou and Lindsay, 2009), and is predicted to be involved in inflammatory and immunological responses similar to how numerous other miRNAs have been implicated in the pathogenesis of PD (Mo et al., 2017). A previous study has shown that hsa-miR-626 is a possible target of leucine-rich repeat kinase 2 (LRRK2), which is involved in the pathogenesis of PD, and is down-regulated in PD plasma (Khoo et al., 2012). Qin et al. (2019) concluded in their study that the expression level of hsa-miR-626 was significantly reduced in the CSF of patients with PD, and suggested this could be a potential diagnostic biomarker for PD. With in-depth studies of CSF miRNAs in patients with PD, these are promising biomarkers for evaluating disease progression and treatment efficacy.

### Circulating LncRNAs and PD

Long non-coding RNA is a type of RNA with a length of 200–100,000 nt that does not code protein. It has a specific secondary structure, as well as being tissue-specific, and shows spatiotemporal specificity in its expression. The tissue specificity of lncRNA is much stronger than that of protein-coding RNA. Long non-coding RNAs not only have different expression levels in different tissues, but also have different expression patterns in different parts of the same tissue (Cabili et al., 2011; Han et al., 2012; Zhao et al., 2015). In addition, lncRNA shows strong spatiotemporal specificity, with the expression level of the same lncRNA significantly different at different stages of development in the same tissue or organ (Kim et al., 2015; Wang et al., 2015). It has been shown that lncRNA plays an important role in the regulation of gene expression, cell proliferation, migration, and apoptosis at transcriptional, post-transcriptional and epigenetic levels (Yoon et al., 2012; Adelman and Egan, 2017). LncRNAs are abundant in the mammalian brain and mainly maintain brain growth, development and function, including neuronal growth and differentiation, synapse formation and maintenance, learning and cognition, memory and other processes (Li et al., 2018c). In recent years, the abnormal expression of lncRNAs in patients with PD suggests that they may play an important role in the development of this disease. The antisense transcript lncRNA, AS Uchl1, was down-regulated in PD rats. AS Uchl1 promoted the expression of Uchl1 protein by regulating Uchl1 mRNA, while down-regulated AS Uchl1 expression induced PD disease progression (Carrieri et al., 2015). LncRNA AS microtubule-associated protein tau (MAPT) is a product of the antisense transcription of a gene encoded by the *MAPT*, which can methylate the *MAPT* promoter to further downregulate MAPT expression. Coupland et al. (2016) found that AS MAPT in PD can regulate tau protein expression at the post-transcriptional level. AS MAPT expression was significantly reduced in PD and may be related to the development of PD (Coupland et al., 2016). Metastasis-associated lung adenocarcinoma transcript 1 (Malat1), located on chromosome 11q13.1, is about 6.7 kb in length and is a highly conserved lncRNA. Kraus et al. (2017) found that Malat1 was up-regulated threefold in patients with PD (Kraus et al., 2017). In a PD mouse model, Malat1 binds miR-129 and down-regulates its expression, thereby eliminating the inhibition of *SNCA* gene expression by miR-129. This suggests that the Malat1/miR-129/SCNA pathway plays an important role in PD development (Xia et al., 2019). HOX antisense intergenic RNA (HOTAIR) is located on human chromosome 12q13.13 (Yang et al., 2011). Current research indicates that HOTAIR is up-regulated in various diseases and is involved in regulating cell differentiation and apoptosis (Liu et al., 2018). Wang et al. (2017) found that HOTAIR expression was up-regulated in PD mouse models. HOTAIR regulated gene expression at the post-transcriptional level. For example, it was related to enhancing LRRK2 mRNA stability and promoting dopaminergic neuron apoptosis. Silencing HOTAIR inhibits caspase-3 activation and neuronal apoptosis (Wang et al., 2017).

Circulating lncRNAs were also described that could be used to evaluate the occurrence and development of PD (Figure 1). In a lncRNA expression profile analysis, Wang Q. et al. (2019) enrolled 32 patients with PD and 13 healthy controls. By using next-generation sequencing, they identified 15 up-regulated and 24 down-regulated lncRNAs in patients with PD. Of the up-regulated lncRNAs, MSTRG.242001.1 and MSTRG.169261.1 were highly expressed among PD patients. Two down-regulated lncRNAs, lnc-MKRN2-42:1 and GAS5:46, were determined by qRT–PCR. Further analysis showed lnc-MKRN2-42:1 expression was positively associated with PD severity (evaluated by MDS-UPDRS score) (Wang Q. et al., 2019). Another study was undertaken on differentially expressed lncRNAs in the CSF of 27 PD patients and 30 healthy controls. Using genome mapping analysis, two lncRNAs (AC079630 and UC001lva.4 located on the *LRRK2* locus), were found to be down-regulated in the CSF of patients with PD compared to healthy controls (Hossein-Nezhad et al., 2016). In short, lncRNAs in the circulating fluids of PD patients need further research in order to identify new biomarkers for the development of PD.

## Current Challenges and Prospects

With the development of technology and in-depth research on miRNAs/lncRNAs, non-coding RNAs (ncRNAs), which were previously thought to have no biological function, have now been found to participate in the regulation of important cellular activities, such as cell growth, proliferation, differentiation, and homeostasis, in various ways. These have a close relationship with the occurrence and development of neurodegenerative diseases, and have become new research hotspots. MicroRNAs/lncRNAs not only provide a new perspective for the understanding of disease mechanisms, but also provide a new method for the diagnosis and treatment of disease. Compared with genes encoding proteins, miRNA/lncRNA expression is more tissue- and spatiotemporal specific. Therefore, these might be used as biomarkers and therapeutic targets of disease, and have an important application in the diagnosis of PD. However, circulating miRNAs have not yet reached specificity and sensitivity standards as clinical biomarkers, so they have not been applied in clinical practice. It is worth pointing out that current research on miRNAs/lncRNAs and the diagnosis of PD is still immature, and conclusions are not robust. Until recently, circulating miRNAs in the field of CNS diseases have begun to be explored since they may become very valuable biomarkers in the early stages. In addition, circulating miRNAs may help reveal the complex pathophysiological mechanisms of CNS diseases. However, many factors affect the expression level of circulating miRNAs, which should be considered. In addition, the types of body fluid used to measure circulating miRNAs is very important since CSF, plasma, and serum samples show inconsistencies in the expression direction of circulating miRNAs. Therefore, larger sample sizes and specialized studies to verify circulating miRNAs in PD are needed. In addition, methodological and technical issues need to be considered, such the choice of a sequencing platform (qRT–PCR vs. NGS), sample separation and purification, and data standardization. For example, (1) applying standard operating procedures for separation and processing; (2) focusing on circulating miRNA fingerprints instead of individual circulating miRNAs; and (3) establishing an experiment verification study. Moreover, how to select efficient and specific miRNAs/lncRNAs and apply them to a clinical diagnosis will be a major problem facing researchers. The solution to this will require not only an improvement in research methods and technologies, but must also include large consortia and repositories that house prospective clinical and biological samples from very large cohorts of patients. In addition, the following future research has been suggested: (1) to further clarify the role of miRNA/lncRNAs in the pathogenesis of PD, which is the basis for selecting target miRNA/lncRNAs; and (2) to optimize the processing and detection methods of miRNA/lncRNAs in experiments. How to promote the dissociation of ncRNAs and protein factors is the key to an accurate quantification of ncRNAs. Further development of the field of circulating miRNAs is required, which may lead to the early diagnosis of PD as well as an evaluation of the prognosis of specific treatments. Nevertheless, improvements in technical and methodological approaches are essential before circulating miRNAs can became clinical biomarkers.

## Conclusion

Due to the limited detection methods available in the clinic, a diagnosis of PD has been very difficult to make with any accuracy. MiRNAs/lncRNAs play an important role in the pathogenesis of PD. Therefore, increasing studies have begun to pay attention to the value of miRNAs/lncRNAs in the diagnosis of PD, and to explore clinical diagnostic methods for using miRNAs/lncRNAs in blood and CSF as biomarkers of PD. With the continuous publication of research results, it is becoming increasingly possible to select specific miRNAs/lncRNAs as biomarkers for a clinical diagnosis of PD. It is necessary to further study and clarify the regulatory mechanisms of miRNAs/lncRNAs in PD so as to improve the applicability and accuracy of these as biomarkers for clinical diagnoses. At the same time, as a potential drug target, miRNAs/lncRNAs may play a greater role in the treatment of PD.

## Author Contributions

YY, YL, and HY wrote this manuscript. JG produced the figure. NL supervised the work. All authors read and approved the final manuscript.

## Conflict of Interest

The authors declare that the research was conducted in the absence of any commercial or financial relationships that could be construed as a potential conflict of interest.
